# Long non-coding RNA cancer susceptibility candidate 2 regulates the function of human fibroblast-like synoviocytes via the microRNA-18a-5p/B-cell translocation gene 3 signaling axis in rheumatoid arthritis

**DOI:** 10.1080/21655979.2021.2022075

**Published:** 2022-01-19

**Authors:** Zhiqin Ye, Lu Wei, Xietian Yin, Huiling Li, Guifu Qin, Siqi Li, Tingting Peng, Bo Liu, Shichao Zhao, Qin Zhuo

**Affiliations:** a Department of Rheumatism Immunology, Hubei Provincial Hospital of Traditional Chinese Medicine, Wuhan, China; b Affiliated Hospital of Hubei University of Chinese Medicine, Hubei Province, Wuhan, China; c Hubei Institute of Traditional Chinese Medicine, Wuhan, Hubei Province, China; d College of the First Clinical, Hubei University of Traditional Chinese Medicine, Wuhan, China

**Keywords:** Rheumatoid arthritis, human fibroblast-like synoviocytes, long non-coding RNA cancer susceptibility candidate 2, microRNA-18a-5p, B-cell translocation gene 3

## Abstract

Rheumatoid arthritis (RA) is a perennial inflammatory condition. Preliminary research indicated that long non-coding (lnc)RNA cancer susceptibility candidate 2 (CASC2) was downregulated in the serum of RA patients. Our study was designed to reveal the roles of lncRNA CASC2 in RA and the latent mechanisms underlying its role. Bioinformatics method (Starbase) and dual-luciferase reporter assay revealed that microRNA (miR)-18a-5p directly interacted with lncRNA CASC2. Furthermore, lncRNA CASC2 and miR-18a-5p expression in the serum samples of RA patients and healthy controls were measured via reverse transcription-quantitative PCR. Compared with the healthy subjects, lncRNA CASC2 was downregulated, whereas miR-18a-5p was upregulated in patients with RA. Overexpression of lncRNA CASC2 decreased the viability of human fibroblast-like synoviocytes (HFLSs) and induced apoptosis, as revealed by the MTT (3-(4,5-dimethylthiazol-2-yl)-2,5-diphenyltetrazolium bromide) assay and flow cytometry analyses. Furthermore, the Western blotting assay suggested that Bax was upregulated and Bcl-2 was downregulated in lncRNA CASC2 up-regulated HFLSs. Downregulation of tumor necrosis factor alpha (TNF-α), interleukin (IL)-1β, IL-6, matrix metalloproteinase (MMP)1, and MMP3 levels by lncRNA CASC2 up-regulation was determined using enzyme-linked immunosorbent assays (ELISAs). However, HFLSs co-transfected with miR-18a-5p mimic exhibited opposite effects compared with the case for the overexpression of lncRNA CASC2. The aforementioned methods were used to verify that a binding site exists between B-cell translocation gene 3 (BTG3) and miR-18a-5p. The effects of miR-18a-5p inhibitor on HFLSs were reversed by BTG3 silencing. Overall, lncRNA CASC2 alleviated RA by adjusting the miR-18a-5p/BTG3 signaling axis and could serve as a novel therapeutic option for RA.

## Introduction

Rheumatoid arthritis (RA) is a chronic joint disease. The main symptoms are persistent synovitis and systemic inflammation [1]. The etiology and pathogenesis remain unclear [2], although novel biological therapies and theories continuously emerge. Human fibroblast-like synoviocytes (HFLSs) play a significant role in the pathogenesis of RA [3]. They promote intimal hyperplasia and overexpress cytokines and chemokines in the process of acquiring a tumor-like aggressive phenotype during the inflammatory response [4]. The level of MMPs and cathepsins is upregulated, which eventually leads to cartilage destruction during RA [5–7].

Following notable developments in the elucidation of RA epigenetics, relevant research on long non-coding (lnc)RNA/microRNA (miRNA/miR) networks has attracted increasing attention [8,9]. miR-4423-3p has been reported to be a novel strategy for the treatment of RA [10]. It has been reported that long intergenic non-protein-coding RNA162 (LINC00162) changes in epigenetic mechanisms, such as the expression of pro-inflammatory cytokines and cell invasion, by secreting mir-4701-5p in RA HFLSs [11]. LncRNA maternally expressed 3 (MEG3) alleviated RA by deactivating the AKT/mTOR signaling pathway, mediated by miR-141 [12]. LncRNA cancer susceptibility candidate 2 (CASC2) is a stable biomarker in RA. LncRNA CASC2 was found to be downregulated in the plasma of RA patients compared with its levels in the plasma of healthy controls, and lncRNA CASC2 overexpression could induce more apoptotic HFLSs by inhibiting IL-17 expression [13]. Currently, the underlying mechanism of lncRNA CASC2 in RA remains largely unclear and requires further study. Based on these prospects, it could become a new treatment strategy for RA. This study will further determine the effect of lncRNA CASC2 on the molecular mechanism of RA in HFLSs.

Through bioinformatics analysis, we found that lncRNA CASC2 may have a targeting relationship with miR-18a-5p, and B-cell translocation gene 3 (BTG3) is a potential target gene of miR-18a-5p. Thus, miR-18a-5p and BTG3 may be abnormally expressed in the plasma of RA patients, and participate in the regulatory mechanism of lncRNACASC2 on RA. As previous study [13] suggested that lncRNA CASC2 may participate in RA by regulating the physiology of HFLSs. And HFLSs are widely used for *in vitro* studies of RA [10,11]. Therefore, this study investigated the role of lncRNA CASC2 in RA by using HFLSs.

In this study, we hypothesized the following: i) lncRNA CASC2/miR-18a-5p/BTG3 are abnormally expressed in the serum of RA patients; ii) lncRNA CASC2 regulates the progression of RA; and iii) the potential mechanisms of lncRNA CASC2 are associated with the miR-18a-5p/BTG3 signaling axis. Therefore, our study was designed to explore the role of lncRNA CASC2 in RA and to illustrate its latent mechanisms in regulating the miR-18a-5p/ B-cell translocation gene 3 (BTG3) signaling axis.

## Materials and methods

### Clinical samples

Serum was obtained from 30 patients with RA, and serum of 30 healthy volunteers was used as the control. All specimens were rapidly frozen, stored in liquid nitrogen, and preserved at −80°C until subsequent experiments. Their clinical characteristics of RA patients were summarized in Table 1. RA patients were diagnosed according to the 2010 American College of Rheumatology (ACR)/European League against Rheumatism (EULAR) criteria for the classification of RA [14]. Exclusion criteria: patients with kidney, liver disease, inflammatory disease, diabetes, tumors and other autoimmune diseases. All patients signed informed consent and agreed to the use of their serum in this study. This study was approved by the Ethics Committee of Hubei Provincial Hospital of Traditional Chinese Medicine (Hubei, China).

**Table 1.** Clinical characteristics of RA patients

Parameters of patients RA patients (*n* = 30) Healthy controls (*n* = 30)	
Age, years, median (range)	58 (32–76) 56 (29–74)
Sex, Female/Male	19/11 18/12
Disease duration, years, median (range)	8 (2–24) –
BMI median (range)	24 (18–25) 23(18–25)

### Cell culture

HFLSs from 3 patients with RA (belong to the 30 patients mentioned above) were separated as previously described [11]. This study was approved by the Ethics Committee of Hubei Provincial Hospital of Traditional Chinese Medicine (Hubei, China). A tissue block culture method was used to isolate the HFLSs form synovial tissues [11]. In brief, the synovial tissue was dissected, fat, blood vessels and fibrous tissue were removed, washed with phosphate buffered saline (PBS), minced into about 1 mm^3^ size, and transferred to a culture flask. For tissue adherence, the culture flasks, which contained Dulbecco’s modified Eagle’s medium (DMEM, Gibco, USA) with 10% fetal bovine serum (FBS, Gibco, USA), were placed upright in an incubator 37°C with 5% CO_2_. After 5 hours, place the flask carefully flat, and change the medium every 2 days. After HFLSs migrated from the tissue explants and grew into a monolayer of firmly adherent cells with a confluence of 90%-95%, HFLSs were trypsinized and collected. Then, the cells were cultivated in DMEM (Gibco, USA) containing 15% fetal bovine serum (FBS, Gibco), 100 mg/ml streptomycin, and 100 U/ml penicillin in a humidified incubator with 5% CO_2_ at 37°C. HFLS passaged 3–5 times were used in the following experiments.

### Cell transfection

For lncRNA CASC2 over-expression, lncRNA CASC2 plasmid was used. The lncRNA CASC2 sequence was synthesized based on the lncRNA CASC2 sequence and then sub-cloned into the pcDNA3.1 vector (lncRNA CASC2-plasmid; Shanghai GeneChem Co., Ltd.). The empty pcDNA3.1 vector was used as a control (control plasmid). The control plasmid, lncRNA CASC2 plasmid, mimic control (cat. no. miR1N0000001-1-5; Guangzhou RiboBio Co., Ltd., Guangzhou, China), miR-18a-5p mimic (cat. no. miR10000072-1-5; Guangzhou RiboBio Co., Ltd., Guangzhou, China), inhibitor control (cat. no. miR2N0000001-1-5; Guangzhou RiboBio Co., Ltd., Guangzhou, China), miR-18a-5p inhibitor (cat. no. miR20000072-1-5; Guangzhou RiboBio Co., Ltd., Guangzhou, China), control small interfering (si)RNA, and BTG3 siRNA (Santa Cruz Biotechnology, Inc.) were transfected into HFLSs using Lipofectamine 2000 (Invitrogen; Thermo Fisher Scientific, Inc.) for 48 h referring to the manufacturer’s protocol. Subsequently, RNA was extracted for qRT-PCR analysis, and Western blotting was adapted to evaluate protein expression.

### *Dual-luciferase reporter assay* [15]

Bioinformatics analysis (StarBase) was applied to investigate the potential target genes of lncRNA CASC2. We predicted the binding sites between miR-18a-5p and lncRNA CASC2. To confirm the binding sites between lncRNA CASC2 and miR-18a-5p, dual-luciferase reporter assay was performed. The 3’UTR of lncRNA CASC2, which contains the miR-18a-5p binding site or mutated target site, was synthesized by genomic PCR and cloned into pMIR vectors (Ambion, USA) to construct the reporter vector lncRNA CASC2 wild-type (lncRNA CASC2-WT) or lncRNA CASC2 mutated-type (lncRNA CASC2-MUT). The 293 T cells (5 × 10^4^ cells per well; 24 well plates) were transfected with the lncRNA CASC2 wild-type or the mutant portion combined with lncRNA CASC2, and the 100 nM miR-18a-5p mimic (cat. no. miR10000072-1-5; Guangzhou RiboBio Co., Ltd., Guangzhou, China) or 100 nM mimic control (cat. no. miR1N0000001-1-5; Guangzhou RiboBio Co., Ltd., Guangzhou, China) by Lipofectamine 2000 (Invitrogen; Thermo Fisher Scientific, Inc.). They were incubated for 48 h following the manufacturer’s protocol. The Dual-Luciferase Reporter Assay System (Promega) was applied to evaluate luciferase activity.

TargetScan was used to predict the binding sites between miR-18a-5p and BTG3, and dual-luciferase reporter assay was used to confirm the binding sites. In experiments confirming the binding of miR-18a-5p and BTG3, the fragment of BTG3, including the binding sites with miR-18a-5p, as well as fragments with mutant (MUT) miR-18a-5p-binding sites, were cloned into pMIR vectors (Ambion, USA). Next, 293 T cells were co-transfected with 100 nM miR-18a-5p mimic (cat. no. miR10000072-1-5; Guangzhou RiboBio Co., Ltd., Guangzhou, China) or 100 nM mimic control (cat. no. miR1N0000001-1-5; Guangzhou RiboBio Co., Ltd., Guangzhou, China) and luciferase reporter plasmids. 48 h after the cell transfection, the Dual-Luciferase Reporter assay system (Promega Corporation) was used to determine luciferase activity.

### RT-qPCR analysis

After treatment, lncRNA CASC2, miR-18a-5p, BTG3, Bax, Bcl-2, or GAPDH expression levels were measured via RT-qPCR. The RNA isolation from the serum of 30 healthy volunteers and 30 patients with RA was obtained with an RNA-isolation Kit (Life Technologies, USA), referring to the manufacturer’s protocol. To eliminate DNA contamination, the isolated RNA was treated with deoxyribonuclease (DNAse). Next, total RNA (5 μg) was reverse transcribed into cDNA according to the PrimeScript RT Reagent Kit (Takara Biotechnology Co., Ltd.) instructions. The qRT-PCR analysis was conducted using the SYBR PrimeScript RT-PCR Kit (TaKaRa) with the ABI 7500 Real-Time PCR System (Agilent Technologies, USA). Expression of target genes was calculated using the 2^−ΔΔCt^ method [16]. Primer sequences were listed as following:

GAPDH forward, 5’-CATCATCCCTGCCTCTACTGG-3’;

reverse, 5’-GTGGGTGTCGCTGTTGAAGTC-3’;

U6 S, 5’-GGAACGATACAGAGAAGATTAGC-3’;

Stem-loop-R, 5’-CTCAACTGGTGTCGTGGAGTC-3’;

lncRNA CASC2 forward, 5’-GCACATTGGACGGTGTTTCC-3’;

reverse, 5’-CCCAGTCCTTCACAGGTCAC-3’;

miR-18a-5p forward, 5’-UAAGGUGCAUCUAGUGCAGAUAG-3’;

reverse, 5’-CUAUCUGCACUAGAUGCACCUUA-3’;

BTG3 forward, 5’-CTCCTCCTGTTCCATTTGGT-3’;

reverse, 5’-TAATCCAGTGATTCCGGTCA-3’;

Bcl-2 forward, 5’-AGGATTGTGGCCTTCTTTGAG-3’;

reverse, 5’-AGCCAGGAGAAATCAAACAGAG-3’;

Bax forward, 5’-TCTGAGCAGATCATGAAGACAGG-3’;

reverse, 5’-ATCCTCTGCAGCTCCATGTTAC-3’.

*MTT assay* [17]. After treatment, HFLSs (4x10^3^ cells/well) were cultured in 96-well plates at 37°C. Then, cells were treated with 10 μl MTT (5 mg/ml) solution and continuously incubated for an additional 4 h at 37°C. The culture medium was removed, and 150 µl of dimethyl sulfoxide (DMSO) was added to dissolve the formazan product in darkness for 10 min. Finally, the OD_570_ was measured using a microplate reader (BioTek, USA) after vibration mixing, according to the manufacturer’s protocol.

### *Flow cytometry analysis* [18]

After treatment for 48 h, HFLSs were detected using a Double-staining Apoptosis Detection Kit (Beyotime Institute of Biotechnology) following the product protocol. Briefly, the cells were incubated with 5 μl Annexin V-FITC and 10 μl propidium iodide at 4°C for 15 min without light. Finally, apoptotic cells (early+late apoptosis) were evaluated using flow cytometry (BD Biosciences, USA) with Kaluza analysis software (version 2.1.1.20653; Beckman Coulter, Inc.).

### *Western blot analysis* [19]

The total proteins were harvested from HFLSs with the RIPA lysis buffer (Beyotime) with protease inhibitors (Beyotime). The protein concentration was determined using a BCA Protein Assay Kit (Invitrogen, USA). Then, protein samples (40 μg protein/lane) were resolved using a 10% SDS-PAGE and transferred to a PVDF membrane. After incubating with 5% skim milk in PBST for 1 h, the membranes were cultured in primary antibodies against GAPDH (1:2,500 dilution, cat. no. ab9485; Abcam), BTG3 (1:1,000 dilution, cat. no. bs-7698 R; Bioss), Bcl-2 (1:1,000 dilution, cat. no. ab32124; Abcam), and Bax (1:1,000 dilution, cat. no. ab182734; Abcam) overnight at 4°C. After washing in TBST, the membranes were treated with secondary antibody (1:2,000 dilution, cat. no. ab96899, Abcam)for 1 h. Finally, the protein bands were visualized by enhanced chemiluminescence detection system reagents (Pierce, USA) and quantified using ImageJ software.

### *ELISAs* [20]

After treatment for 48 h, HFLSs were harvested and centrifuged for 10 min at 400 × *g*, and inflammatory factor levels of IL-6 (cat. no. ab178013), IL-1β (cat. no. ab214025), TNF-α (cat. no. ab181421), MMP1 (cat. no. ab215083), and MMP3 (cat. no. ab269371) in the HFLS supernatant were quantified using ELISA kits (Abcam) following the manufacturer’s protocols. The OD value of the samples in each well was determined at 450 nm by a Multiscan Spectrum Microplate Reader (Thermo scientific), following the manufacturer’s protocol.

### Statistical analysis

All experiments were repeated at least three times. Statistics are presented as the mean ± SD, and data were analyzed using SPSS 19.0 statistical software (IBM Corp.). Unpaired Student’s t-tests or one-way ANOVAs followed by post hoc Tukey’s test were applied for determining differences between groups. We considered P < 0.05 as statistically significant.

## Results

**Figure 1.**
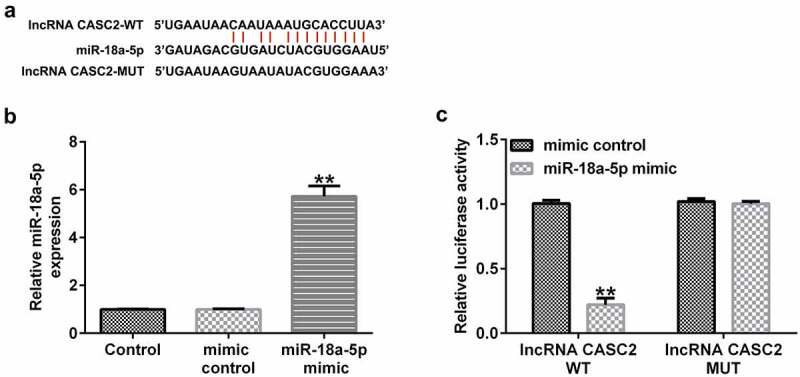
miR-18a-5p binds to the 3’UTR of lncRNA CASC2. (a) Prediction of a complementary lncRNA CASC2 and miR-18a-5p binding site. (b) Expression of miR-18a-5p in 293 T cells after mimic control or miR-18a-5p mimic transfection. (c) The Dual-luciferase reporter assay confirmed the relationship between lncRNA CASC2 and miR-18a-5p. **P < 0.01 vs. mimic control. miR, microRNA; lncRNA CASC2, long non-coding RNA cancer susceptibility candidate 2. Experiments were repeated for three times.

*miR-18a-5p is targeted by lncRNA CASC2*. We firstly confirmed the relationship between miR-18a-5p and lncRNA CASC2. It was predicted that miR-18a-5p was a latent target of lncRNA CASC2 using the bioinformatics software, StarBase (Figure 1(a)). Subsequently, a dual-luciferase reporter assay was performed to confirm the binding sites between miR-18a-5p and lncRNA CASC2. Firstly, we confirmed the transfection efficiency of miR-18a-5p mimic in 293 T cells, and the data revealed that compared to the mimic control group, the miR-18a-5p mimic significantly enhanced miR-18a-5p levels in 293 T cells (Figure 1(b)). As shown in Figure 1(c), dual-luciferase reporter assay results indicated that the miR-18a-5p mimic dramatically decreased luciferase activity of lncRNA CASC2-WT, but not lncRNA CASC2-MUT, indicating that miR-18a-5p is targeted by lncRNA CASC2.

**Figure 2.**
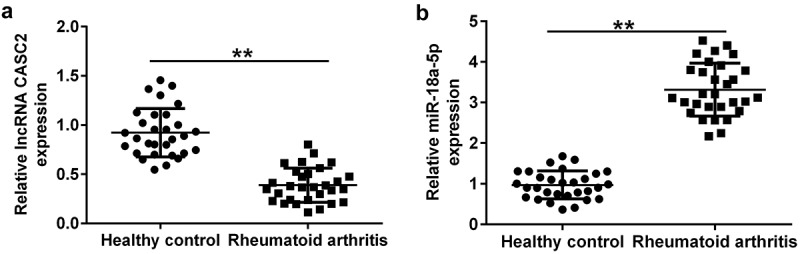
Level of lncRNA CASC2 and miR-18a-5p in the sera of patients with RA and healthy volunteers. Reverse transcription-quantitative PCR analysis of (a) lncRNA CASC2 and (b) miR-18a-5p levels in the sera of patients with RA and healthy volunteers. **P < 0.01 vs. healthy control. miR, microRNA; lncRNA CASC2, long non-coding RNA cancer susceptibility candidate 2; RA, rheumatoid arthritis. Experiments were repeated for three times.

*lncRNA CASC2 is downregulated, and miR-18a-5p is overexpressed in the sera of RA patients*. To determine the expression of miR-18a-5p and lncRNA CASC2 in RA, RT-qPCR was performed. Levels of lncRNA CASC2 and miR-18a-5p in the serum of 30 cases of RA patients and 30 cases of healthy controls were determined using RT-qPCR. Compared to the healthy control group, lncRNA CASC2 was confirmed to be downregulated (Figure 2(a)), whereas miR-18a-5p was upregulated in the sera of RA patients (Figure 2(b)).

### lncRNA CASC2 influences HFLS physiology by regulating miR-18a-5p expression

**Figure 3.**
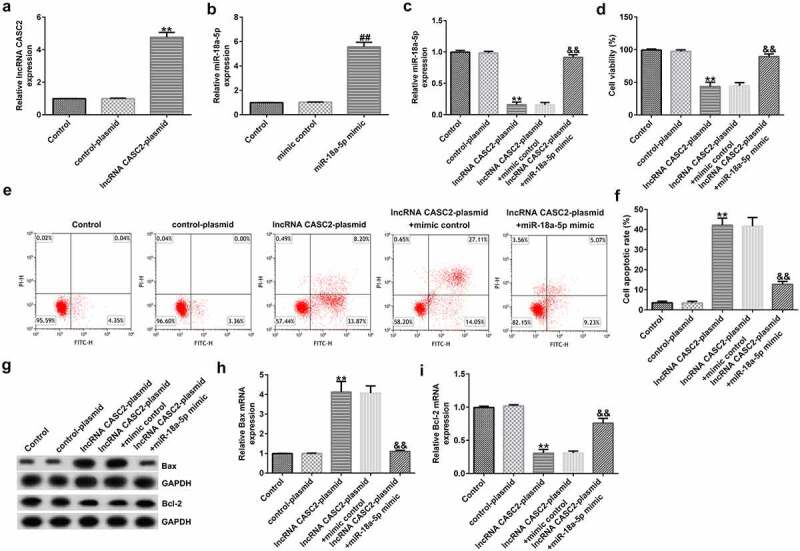
miR-18a-5p mimic transfection reverses the effects of lncRNA CASC2 on HFLS proliferation and apoptosis. Control plasmids or lncRNA CASC2 plasmids were transfected into HFLSs. (a) mRNA expression of lncRNA CASC2 in HFLSs. (b) RT-qPCR analysis of miR-18a-5p expression in mimic control- or miR-18a-5p mimic-transfected HFLSs. (c) Determination of miR-18a-5p levels in HFLSs transfected with the lncRNA CASC2 plasmid + mimic control or lncRNA CASC2 plasmid + miR-18a-5p mimic using RT-qPCR. (d) MTT analysis of the viability of HFLSs. (e) Apoptotic cells were analyzed using flow cytometry. (f) Quantification of apoptotic cells. (g) Determination of Bax and Bcl-2 proteins using Western blot assay. (h) Bax mRNA levels in different groups. (i) Bcl-2 mRNA levels in different groups. **P < 0.01 vs. control plasmid; ##P < 0.01 vs. mimic control; &&P < 0.01 vs. lncRNA CASC2 plasmid + mimic control. miR, microRNA; lncRNA CASC2, long non-coding RNA cancer susceptibility candidate 2; RT-qPCR, reverse transcription-quantitative PCR; HFLSs, human fibroblast-like synoviocytes. Experiments were repeated for three times.

To further illustrate the biological behavior of lncRNA CASC2 in the HFLSs, the control plasmid, lncRNA CASC2 plasmid, mimic control, and miR-18a-5p mimic, lncRNA CASC2 plasmid+mimic control, or lncRNA CASC2 plasmid+miR-18a-5p mimic were transfected into the HFLSs. Furthermore, RT-qPCR analysis revealed that lncRNA CASC2 plasmid- and miR-18a-5p mimic-transfected cells expressed significantly higher lncRNA CASC2 (Figure 3(a)) and miR-18a-5p (Figure 3(b)) levels compared to the control cells. Moreover, compared to the control plasmid group, upregulation of lncRNA CASC2 reduced the miR-18a-5p levels, the effect of which was reversed in HFLSs transfected with the miR-18a-5p mimic (Figure 3(c)).

**Figure 4.**
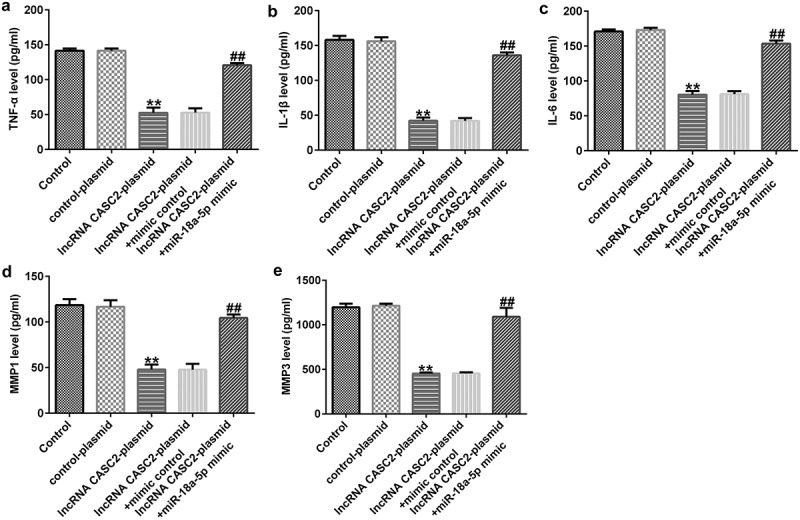
miR-18a-5p mimic abrogates lncRNA CASC2 effects on inflammatory factor release in HFLSs. After transfection, the excretion of inflammatory cytokines, including (a) TNF-α, (b) IL-1β, and (c) IL-6, and MMPs including MMP1 (d) and MMP3 (e) were analyzed via ELISAs in different groups. **P < 0.01 vs. control plasmid; ##P < 0.01 vs. lncRNA CASC2 plasmid + mimic control. miR, microRNA; lncRNA CASC2, long non-coding RNA cancer susceptibility candidate 2. Experiments were repeated for three times.

As displayed in Figure 3(d-e), compared to the control plasmid, the lncRNA CASC2 plasmid transfection significantly reduced the viability of HFLSs and promoted cell apoptosis. Biomarkers of apoptosis were detected via Western blot and RT-qPCR analysis, indicating that the protein and mRNA levels of Bax were elevated, whereas the Bcl-2 protein and mRNA levels were dramatically inhibited after transfection with the lncRNA CASC2 plasmid. Inflammation of HFLSs may be attenuated because the release of inflammatory factors (TNF-α, IL-1β, and IL-6) and MMPs (MMP1 and MMP3) was notably lower following transfection with the lncRNA CASC2 plasmid (Figure 4(a-e)). Co-transfection with the miR-18a-5p mimic significantly reversed all these changes.

**Figure 5.**
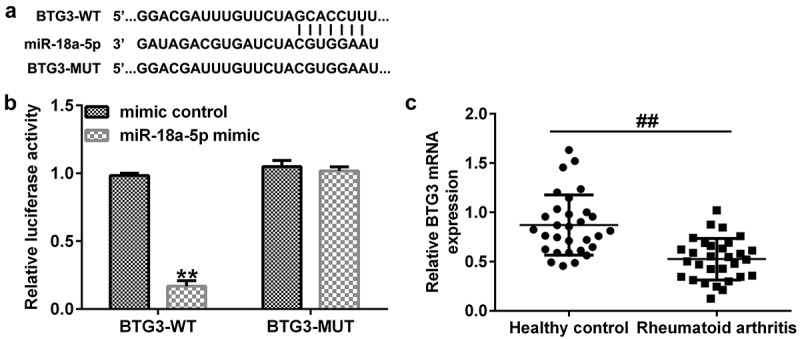
BTG3 directly targets miR-18a-5p. (a) A schematic of the miR-18a-5p binding site in lncRNA CASC2 3’-UTR. (b) Binding between miR-18a-5p and BTG3 was confirmed using dual-luciferase reporter analysis. (c) mRNA expression of BTG3 in the sera of patients with RA and healthy volunteers. **P < 0.01 vs. mimic control; ##P < 0.01 vs. healthy control. miR, microRNA; lncRNA CASC2, long non-coding RNA cancer susceptibility candidate 2; RA, rheumatoid arthritis; BTG3, B-cell translocation gene 3. Experiments were repeated for three times.

*BTG3 is directly regulated by miR-18a-5p*. To investigate the roles of miR-18a-5p in RA, StarBase was applied to search for the target gene. It was suggested that BTG3 was a potential target of miR-18a-5p (Figure 5(a)). A subsequent dual-luciferase reporter assay further confirmed the binding sequence (Figure 5(b)).

An RT-qPCR assay revealed that the mRNA level of BTG3 was lower in the sera of RA patients than that of healthy volunteers (Figure 5(c)).

**Figure 6.**
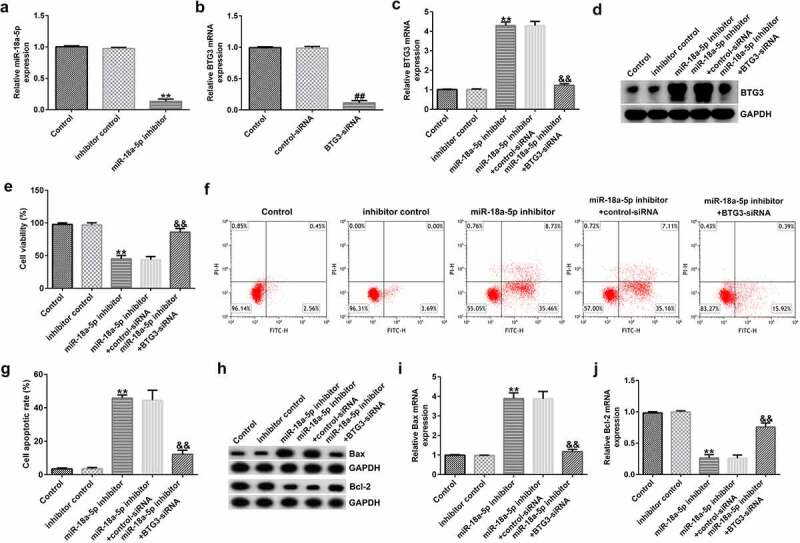
BTG3 siRNA abolishes the influences of the miR-18a-5p inhibitor on HFLSs proliferation and apoptosis. The inhibitor control, miR-18a-5p inhibitor, control siRNA, and BTG3 siRNA were transfected into HFLSs for 48 h. (a) miR-18a-5p expression was evaluated in HFLSs. (b) RT-qPCR analysis of BTG3 in control siRNA- or BTG3 siRNA-transfected HFLSs. Evaluation of BTG3 (c) mRNA and (d) protein levels in transfected HFLSs using RT-qPCR and Western blotting. (e) HFLS viability was measured using an MTT assay. (f) Flow cytometric analysis of apoptotic cells in HFLSs. (g) Quantification of apoptotic cells. (h) Determination of Bax and Bcl-2 protein expression levels using Western blotting. qRT-PCR analysis of Bax (i) and Bcl-2 (j) mRNA levels in different groups. **P < 0.01 vs. inhibitor control; ##P < 0.01 vs. control siRNA; &&P < 0.01 vs. miR-18a-5p inhibitor + control siRNA. miR, microRNA; lncRNA CASC2, long non-coding RNA cancer susceptibility candidate 2; RA, rheumatoid arthritis; BTG3, B-cell translocation gene 3; RT-qPCR, reverse transcription-quantitative PCR; HFLSs, human fibroblast-like synoviocytes; siRNA, small interfering RNA. Experiments were repeated for three times.

*Knockdown of miR-18a-5p promotes HFLS apoptosis and suppresses inflammation. To explore whether* miR-18a-5p affected HFLSs via regulating BTG3 expression, inhibitor control, miR-18a-5p inhibitor, miR-18a-5p inhibitor+control-siRNA, or miR-18a-5p inhibitor+BTG3-siRNA were transfected into HFLSs for 48 h. Compared to the inhibitor control, miR-18a-5p inhibitor transfection was shown to reduce miR-18a-5p expression in HFLSs via RT-qPCR (Figure 6(a)). Similarly, BTG3 mRNA levels were decreased following BTG3 siRNA transfection than the siRNA group (Figure 6(b)). The miR-18a-5p inhibitor transfection led to a significant increase in BTG3, whereas BTG3 siRNA transfection reversed this effect (Figure 6(c,d)).

The viability of HFLSs decreased after miR-18a-5p inhibitor transfection (Figure 6(e)), whereas the apoptosis of cells increased (Figure 6(f,g)). Bax was upregulated (Figure 6(h,i)) compared to the inhibitor control group, whereas Bcl-2 (Figure 6(h,j)) was down expressed after miR-18a-5p inhibitor transfection. All these changes were significantly reversed following BTG3 siRNA transfection.

**Figure 7.**
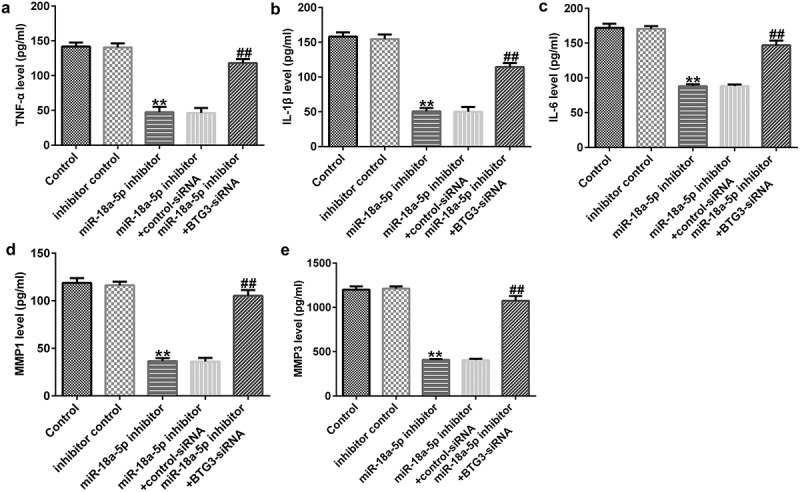
BTG3 siRNA transfection abolishes the miR-18a-5p inhibitor influences on inflammatory cytokine release in HFLSs. After transfection, the secretion of inflammatory cytokines, such as (a) TNF-a, (b) IL-1β, and (c) IL-6, and MMPs including MMP1 (d) and MMP3 (e) were determined via ELISAs in different groups. **P < 0.01 vs. inhibitor control; ##P < 0.01 vs. miR-18a-5p inhibitor + control siRNA. miR, microRNA; BTG3, B-cell translocation gene 3. Experiments were repeated for three times.

To measure the secretion of inflammatory cytokines (TNF-α, IL-1β, and IL-6) and MMPs (MMP1 and MMP3), ELISAs were performed. Our data revealed that miR-18a-5p inhibitor transfection inhibited the levels of TNF-α, IL-1β, IL-6, MMP1, and MMP3 in the supernatant of HFLSs. These decreases were inhibited following BTG3 siRNA transfection (Figure 7(a-e)).

## Discussion

Studies on the mechanism of lncRNA and miRNA, which influence different diseases, including RA, have increased interest levels. A previous study reported the role of lncRNA CASC2 in patients with RA as a potential biomarker and its effect on promoting HFLS apoptosis through downregulating IL-17 [13]. However, the role and mechanism of lncRNA CASC2 in RA are yet to be fully elucidated.

We first confirmed the target relationship between miR-18a-5p and lncRNA CASC2, and the down-regulation of lncRNA CASC2 and up-regulation of miR-18a-5p in the sera of RA patients. A pharmacological treatment could have influenced gene expression. However, the pharmacological treatments of the enrolled patients were not analyzed in this study, thus this was a limitation of this study. We will conduct an in-depth analysis of this in the next research.

As key effector cells, HFLSs contribute to cartilage destruction via the secretion of matrix metalloproteinases and pro-inflammatory factors. The majority of strategies that cure RA target HFLSs [21]. In a previous study, overexpression of lncRNA HOTAIR was shown to inhibit inflammatory cytokine levels, including IL-1β and TNF-α. Through the regulation of miR-138 and inactivation of the NF-κB signaling pathway, lncRNA HOTAIR served as a preserver and improved the symptoms of RA [22]. lncRNA DILC influences RA by inducing apoptosis of HFLSs and downregulating IL-6 expression levels [23]. The miR-222-3p/Sirtuin 1 signaling axis was confirmed as a target of lncRNA growth arrest-specific 5 for regulating inflammation and apoptosis in HFLSs [24].

In our study, further efforts were made to elucidate the molecular mechanism of lncRNA CASC2 in the regulation of HFLSs isolated from patients with RA. The levels of cell apoptosis increased, and the expression of pro-inflammatory cytokines (TNF-α, IL-1β, and IL-6) and MMPs (MMP1 and MMP3) decreased, which indicated the amelioration of RA following lncRNA CASC2 overexpression. Furthermore, miR-18a-5p abolished the effects of lncRNA CASC2 on HFLSs. However, we did not perform analysis to exclude that reduced cytokine level is not simply a consequence of cell death (eg calculation to determine how much of the cytokines are released per alive cells in response to lncRNA CASC2 plasmid etc). This was a limitation of current study. Notably, a binding site was identified between BTG3 and miR-18a-5p. BTG3 is a driver gene for regulating the cell cycle, apoptosis, and invasion in various cancer types [25]. To our knowledge, there have been no previous studies regarding BTG3 participation in RA. Compared to healthy control subject sera, the BTG mRNA levels were notably reduced in RA patients. Moreover, following miR-18a-5p inhibitor transfection, the viability of HFLSs decreased, their apoptosis levels increased, and the expression levels of pro-inflammatory factors in HFLSs were reduced. Moreover, miR-18a-5p was negatively correlated with the expression level of BTG3. Collectively, the results indicated an intact signaling axis between lncRNA CASC2, miR-18a-5p, and BTG3.

Overall, this study was only a preliminary *in vitro* study of the role of lncRNA CASC2 in RA. In the future we will continue to explore the relationship between BTG3 and lncRNA CASC2 in RA. We will conduct a linear regression analysis comparing RA patients serum expression levels of BTG3, lncRNA CASC2 and miR-18a-5p for evidence of relationships. In addition, we will explore the expression, role and interaction of BTG3, lncRNA CASC2 and miR-18a-5p in RA in animal models.

## Conclusion

This study revealed the role of lncRNA CASC2 on the inflammation and apoptosis of HFLSs in RA, the mechanisms underlying these phenomena; the novel regulatory signaling axis, miR-18a-5p/BTG3, may represent a potential target for RA treatment.

## Data Availability

The datasets used and/or analyzed during the current study are available from the corresponding author upon reasonable request.

## Research highlights

lncRNA CASC2 is downregulated, and miR-18a-5p is overexpressed in the sera of RA patients.

lncRNA CASC2 influences HFLS physiology by targeting miR-18a-5p.

Knockdown of miR-18a-5p promotes HFLS apoptosis and suppresses inflammation via BTG3.
